# Apple Pomace Extract Improves MK-801-Induced Memory Impairment in Mice

**DOI:** 10.3390/nu16020194

**Published:** 2024-01-06

**Authors:** Ayako Watanabe, Minori Shimada, Hayato Maeda, Tsuyoshi Narumi, Junji Ichita, Koh Itoku, Akira Nakajima

**Affiliations:** 1 Department of Applied Biology and Food Sciences, Faculty of Agriculture and Life Science, Hirosaki University, 3 Bunkyo-cho, Hirosaki 036-8561, Japanhayatosp@hirosaki-u.ac.jp (H.M.); 2 Department of Industry Development Sciences, Graduate School of Sustainable Community Studies, Hirosaki University, 3 Bunkyo-cho, Hirosaki 036-8561, Japan; 3 Nihon Haruma Co., Ltd., 398 Kanda, Hirosaki 036-8052, Japan; narumi@nihonharuma.com (T.N.); ichita@nihonharuma.com (J.I.); nihon-haruma@beach.ocn.ne.jp (K.I.)

**Keywords:** Alzheimer’s disease, memory, apple pomace extract, RNA sequencing

## Abstract

Alzheimer’s disease (AD) is a neurodegenerative disease that involves progressive cognitive decline accompanied by synaptic degeneration and impaired neurotransmission. Recent studies revealed that apple pomace, a waste byproduct of the apple processing industry, has beneficial health properties, but its potential to prevent and treat AD has not been determined. Herein, we examined the effects of apple pomace extract on *N*-methyl-D-aspartate receptor antagonist MK-801-induced memory impairment in mice. Repeated treatment with apple pomace extract for 7 days reversed the MK-801-induced impairment of associative memory and recognition memory. RNA sequencing revealed that repeated treatment with apple pomace extract altered the gene expression profile in the hippocampus of mice. Real-time PCR showed that apple pomace extract induced upregulation of the mRNA expression for *Zfp125* and *Gstp1*. Furthermore, gene sets related to synapse and neurotransmission were upregulated by apple pomace extract. These findings indicate that apple pomace extract may be useful for the prevention and treatment of AD.

## 1. Introduction

Alzheimer’s disease (AD) is the most common form of dementia and age-related neurodegenerative disease among the elderly [1]. Recent estimates showed that the number of people with dementia would increase from 57.4 million cases worldwide in 2019 to 152.8 million cases in 2050 [2]. AD is characterized by progressive cognitive decline, accompanied by accumulation of amyloid β (Aβ), hyperphosphorylation of tau, synaptic degeneration, and impaired neurotransmission [1,3,4]. Although drugs such as acetylcholinesterase inhibitors (donepezil, galantamine, and rivastigmine) and memantine, a noncompetitive *N*-methyl-D-aspartate (NMDA) receptor modulator, have been used for decades, these are symptomatic treatments and are not able to significantly alter the progression of AD [1]. Recently, it has been demonstrated that lecanemab, the anti-Aβ monoclonal antibody, is a potentially disease-modifying treatment for AD [5]. However, considering its high cost, there are worries that the clinical benefits of anti-Aβ therapy are modest and may only apply to certain highly selected populations [6]. Therefore, the importance of preventive interventions through nutrition and dietary components has been highlighted [7].

Apple pomace, a waste byproduct of apple processing, contains nutritional components such as dietary fiber, minerals, phenolic compounds, carbohydrates, triterpenoids, and essential fatty acids [8,9,10,11]. Preclinical studies have shown that apple pomace and its constituents have beneficial health properties, such as improved lipid metabolism, cardiovascular effects, antioxidant activity, anti-inflammatory activity, and anti-proliferative activity [8,9], demonstrating their potential as functional ingredients in food products.

Concerning the effects of apple pomace and its constituents on neurodegeneration, a recent study showed that apple pomace ameliorated the expression of genes associated with neurodegeneration in the hypothalamus of rats [12]. Isoquercitrin (quercetin-3-O-β-d-glucopyranoside), a constituent of apple pomace, exerted neuroprotective effects in MPTP-induced Parkinson’s disease model mice [13]. However, the effects of apple pomace on memory impairment in an AD model have not been evaluated.

In this study, we investigated the effects of apple pomace extract on NMDA receptor antagonist MK-801-induced memory impairment in mice. Furthermore, we performed an RNA sequencing analysis of the hippocampus in mice treated with apple pomace extract to clarify the mechanisms of action.

## 2. Materials and Methods

### 2.1. Preparation of Apple Pomace Extract

First, 1 kg of dried apple pomace was extracted with 6 L of 80% ethanol for 3 h at 50 °C. After filtration, the filtrate was concentrated in vacuo at 50 °C using a rotary evaporator, and then 2 L of water was added. The precipitate was separated by suction filtration and dried in vacuo to obtain 15 g of the AF1 fraction of apple pomace extract (APE-AF1).

### 2.2. Animals

Male 8-week-old ddY mice were purchased from Japan SLC, Inc. (Hamamatsu, Japan). A total of 87 mice were used in this study. Animals were housed in cages with free access to food and water under a constant temperature (23 ± 1 °C) and adapted to a 12 hr light–dark regime (light on between 7 a.m. and 7 p.m.).

### 2.3. Drugs and Experimental Design

APE-AF1 was suspended in a 0.5% carboxymethyl cellulose (CMC) solution. MK-801 (Sigma-Aldrich, St. Louis, MO, USA) was dissolved in saline. The experimental design of each experiment is as described below.

#### 2.3.1. Passive Avoidance Task (Single Treatment)

**Figure 1:**
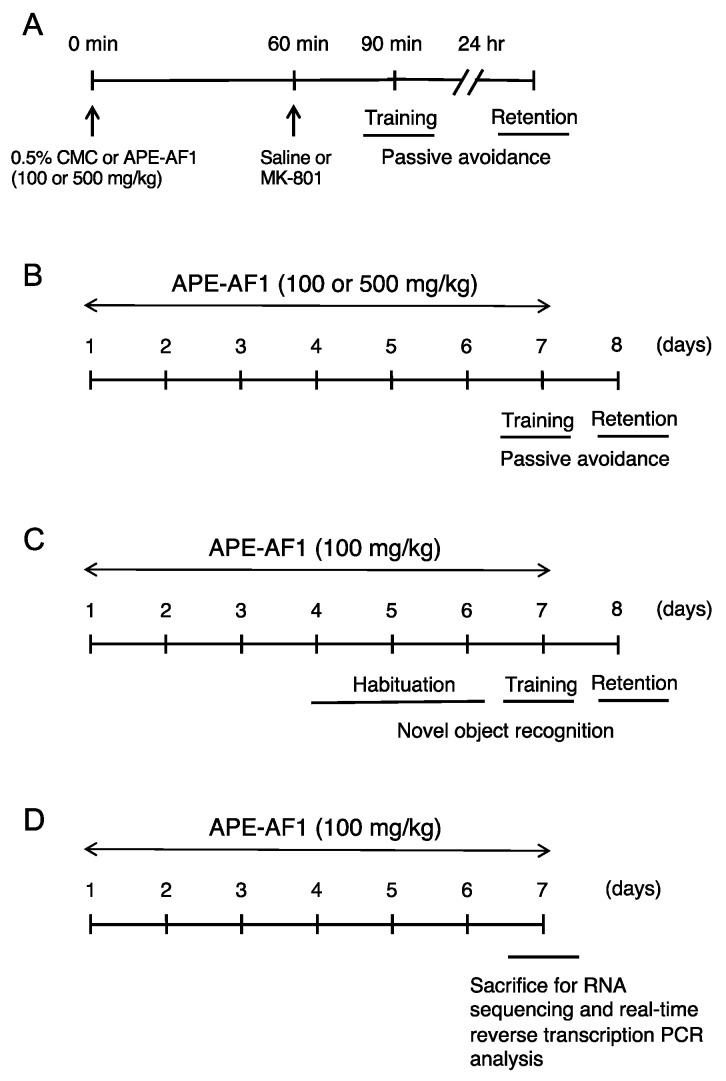
Experimental schedule. (**A**) Passive avoidance task (single treatment). (**B**) Passive avoidance task (repeated treatment). (**C**) Novel object recognition test. (**D**) RNA sequencing and real-time reverse transcription PCR analysis.

The experimental groups to investigate the effects of a single treatment with APE-AF1 in the passive avoidance task were as follows: control, mice treated with saline and 0.5% CMC (n = 5); MK-801, mice treated with MK-801 and 0.5% CMC (n = 5); MK-801+APE-AF1 100 mg/kg, mice treated with MK-801 and 100 mg/kg APE-AF1 (n = 5); and MK-801+APE-AF1 500 mg/kg, mice treated with MK-801 and 500 mg/kg APE-AF1 (n = 5). MK-801 (0.2 mg/kg, s.c.) was given 30 min prior to the training trial. APE-AF1 (100–500 mg/kg, p.o.) or vehicle (0.5% CMC) was administered 60 min prior to MK-801. In the retention test, no drugs were administered. The experimental schedule is shown in Figure 1A.

#### 2.3.2. Passive Avoidance Task (Repeated Treatment)

The experimental groups to investigate the effects of repeated treatment with APE-AF1 in the passive avoidance task were as follows: control, mice treated with saline and 0.5% CMC (n = 10); MK-801, mice treated with MK-801 and 0.5% CMC (n = 10); MK-801+APE-AF1 100 mg/kg, mice treated with MK-801 and 100 mg/kg APE-AF1 (n = 11); and MK-801+APE-AF1 500 mg/kg, mice treated with MK-801 and 500 mg/kg APE-AF1 (n = 9). APE-AF1 (100–500 mg/kg, p.o.) or vehicle (0.5% CMC) was administered once daily for 7 consecutive days (i.e., days 1–7). On day 7, MK-801 (0.2 mg/kg, s.c.) was given 30 min prior to the training trial. APE-AF1 (100–500 mg/kg, p.o.) or vehicle (0.5% CMC) was administered 60 min prior to MK-801. In the retention test on day 8, no drugs were administered. The experimental schedule is shown in Figure 1B.

#### 2.3.3. Novel Object Recognition Test

The experimental groups to investigate the effects of repeated treatment with APE-AF1 in the novel object recognition test were as follows: control, mice treated with saline and 0.5% CMC (n = 7); MK-801, mice treated with MK-801 and 0.5% CMC (n = 7); and MK-801+APE-AF1 100 mg/kg, mice treated with MK-801 and 100 mg/kg APE-AF1 (n = 7). APE-AF1 (100 mg/kg, p.o.) was injected once daily for 7 consecutive days (i.e., days 1–7) because this dosage regimen showed memory-improving effects in the passive avoidance task. On days 4–6, vehicle or APE-AF1 (100 mg/kg p.o.) was injected after habituation. On day 7, vehicle or APE-AF1 (100 mg/kg p.o.) was injected 60 min prior to MK-801, which was administered 30 min before training. During the retention session on day 8, no drugs were administered. The experimental schedule is shown in Figure 1C.

#### 2.3.4. RNA Sequencing and Real-Time Reverse Transcription PCR Analysis

The experimental groups to investigate the effects of APE-AF1 in RNA sequencing and real-time reverse transcription PCR analysis were as follows: control, mice treated with 0.5% CMC (n = 3) and APE-AF1 100 mg/kg, mice treated with 100 mg/kg APE-AF1 (n = 3). Mice were treated with vehicle (0.5% CMC) or APE-AF1 (100 mg/kg, p.o.) once daily for 7 consecutive days. Ninety minutes after the final injection on day 7, mice were sacrificed by cervical dislocation. The hippocampus was isolated and stored at −80 °C until use. The experimental schedule is shown in Figure 1D.

### 2.4. Step-Through Passive Avoidance Task

The step-through passive avoidance task was performed as previously described [14,15] with minor modifications. The training trial and retention test were performed in a Plexiglas box, which contained dark (25 cm × 25 cm × 28 cm) and light (15 cm × 9 cm × 25 cm) compartments. The training trial was started by placing the mouse in the light compartment of the box. An electric shock (0.4 mA, 1 s) was applied to the floor bars once the mouse had fully entered the dark chamber. In the retention test performed 24 h after training, each mouse was put in the light compartment and the step-through latency was measured until 300 s had elapsed.

### 2.5. Novel Object Recognition Test

The novel object recognition test was performed as previously described [15,16] with minor modifications. Mice were individually habituated to an open box (30 cm × 30 cm × 35 cm) for 3 days (i.e., days 4–6). During the training session on the seventh day, two objects were introduced into the box with the mouse, and the time spent exploring each object was recorded for 10 min. During the retention session on the eighth day, the mouse was introduced into the same box in which one of the objects was replaced by a new one. The time spent exploring the two objects was recorded over a 2 min period. The preference index was determined during the retention session (i.e., ratio of time spent exploring the new object to overall time exploring both objects) and used to measure long-term recognition memory.

### 2.6. RNA Sequencing Analysis

An RNA sequencing analysis was performed as previously described [15,17] with minor modifications. Briefly, total RNA was isolated from the hippocampi of 6 mice (i.e., 3 from vehicle and 3 from APE-AF1) using an RNeasy Mini Kit with DNase I. The libraries were sequenced by DNBSEQ-G400 for 2 × 100 base reads. Pair-end clean reads were subsequently mapped to the mouse genome sequence (https://www.ncbi.nlm.nih.gov/assembly/GCF_000001635.27, accessed on 6 December 2022) with Hisat2 (v2.2.1). The counted data were analyzed using the RaNA-Seq cloud platform (https://ranaseq.eu, accessed on 6 May 2023) [18]. The intergroup differences and sample repetition within groups were examined using principal component analysis (PCA). Differentially expressed genes (DEGs) were extracted using DESeq2. Genes with an adjusted *p*-value < 0.05 found by DESeq2 were regarded as differentially expressed. A gene set enrichment analysis (GSEA) was also performed using the RaNA-Seq cloud platform.

### 2.7. Real-Time Reverse Transcription PCR

Real-time reverse transcription PCR was conducted as previously described [15,17]. Complementary DNA (cDNA) was reverse transcribed using the ReverTra Ace (Toyobo, Osaka, Japan). Real-time PCR was performed using an AriaMx instrument (Agilent Technologies, Santa Clara, CA, USA). Supplementary Table S1 displays the primer sequences used in this study. The relative mRNA expression levels were normalized to the β-actin mRNA levels.

### 2.8. Determination of Glucosylceramide in APE-AF1 by HPLC-ESI-Q-TOF-MS

The glucosylceramide composition in APE-AF1 was analyzed via HPLC-ESI-Q-TOF-MS. Chloroform/methanol (2:1, *v*/*v*) was added to APE-AF1 powder in a 10:1 ratio to extract the glucosylceramide fraction. After evaporation of the collected chloroform phase, the residue was saponified with 0.4 mol/L KOH in methanol at 38 °C for 2 h to remove glycerolipids. The alkali-stable fraction was subjected to an HPLC-ESI-TOF-MS analysis.

The sample was analyzed using an HPLC (ACQUITY H-Class system; Waters Corporation, Milford, MA, USA) equipped with a ACQUITY UPLC HSS T3 column (100 mm × 2.1 mm, 1.8 µm particle size; Waters Corporation, MA, USA). The mobile phases were 0.1% formic acid/methanol (3:97, *v*/*v*) with a flow rate of 0.2 mL/min and an injection volume of 3.0 μL. The MS detection was performed in a Xevo G2-XS quadruple-time-of-flight (Q-TOF) (Waters Corporation, MA, USA) mass spectrophotometer equipped with an electrospray ionization source (ESI). The full screen mass spectra detection was carried out in the positive ion mode in a mass range from 100 to 1200 *m*/*z* with a capillary voltage of 3.0 kV, a cone voltage of 30 eV, and a source temperature of 150 °C. Nitrogen was used as a nebulizing gas at a flow rate of 20 L/h. Comparison of the observed MS spectra with those found in the literature and the MassBank database was the main tool used for identification of glucosylceramide.

### 2.9. Statistical Analyses

The results are expressed as means ± SEM. The data for mRNA expression determined by real-time PCR were analyzed via a two-tailed Student’s *t*-test. Other data were analyzed via a one-way analysis of variance (ANOVA), followed by the Bonferroni test. A level of *p* < 0.05 was considered statistically significant.

## 3. Results

### 3.1. APE-AF1 Reverses MK-801-Induced Associative Memory Impairment in the Passive Avoidance Task

**Figure 2:**
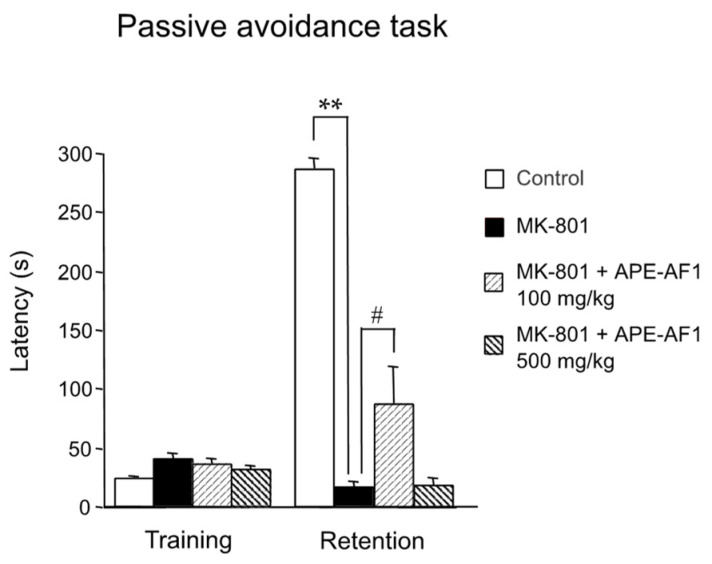
Effects of repeated treatment with apple pomace extract on associative memory in the passive avoidance task. Values of step-through latency are shown as the means ± SEM (n = 9–11). ** *p* < 0.01. # *p* < 0.05.

We first investigated the effects of a single treatment of APE-AF1 (100–500 mg/kg, p.o.) on NMDA receptor antagonist MK-801-induced memory impairment in the passive avoidance task in mice. MK-801 treatment (0.2 mg/kg, s.c.) resulted in a decrease in the step-through latency in the retention test performed 24 h after training (*F*_(3,16)_ = 114.47, *p* < 0.0001). A single treatment with APE-AF1 (100–500 mg/kg, p.o.) had no effects on the step-through latency (Supplementary Figure S1). Next, we examined the effects of repeated treatment with APE-AF1 for 7 days on MK-801-induced memory impairment. Repeated treatment with 100 mg/kg APE-AF1 led to a significant increase in the step-through latency in the retention test, whereas 500 mg/kg APE-AF1 had no effects (*F*_(3,36)_ = 42.499, *p* < 0.0001) (Figure 2). These results suggest that repeated treatment with 100 mg/kg APE-AF1 reversed the MK-801-induced associative memory impairment.

### 3.2. APE-AF1 Reverses the MK-801-Induced Recognition Memory Impairment in the Novel Object Recognition Test

**Figure 3:**
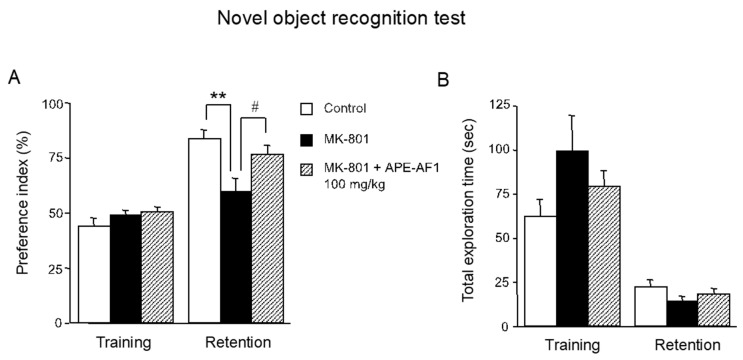
Effects of repeated treatment with apple pomace extract on recognition memory in the novel object recognition test. (**A**) Exploratory preference. (**B**) Total exploration time. Values are shown as the means ± SEM (n = 7). ** *p* < 0.01. # *p* < 0.05.

Next, we examined the effects of APE-AF1 on MK-801-induced impairment of recognition memory. Considering that the repeated treatment with 100 mg/kg APE-AF1 reversed MK-801-induced memory impairment in the passive avoidance task, APE-AF1 (100 mg/kg, p.o.) was injected once daily for 7 consecutive days before the training session of the novel object recognition test. In the training session, there were no differences in exploratory preferences for either object among the three groups (*F*_(2,18)_ = 1.130, *p* = 0.3450) (Figure 3A). In the retention session performed 24 h after training, a decrease in exploratory preference for novel objects was evident in the MK-801-treated group, suggesting impaired discrimination of a novel object from a familiar one (*F*_(2,18)_ = 5.754, *p* = 0.0117) (Figure 3A). Repeated treatment with APE-AF1 (100 mg/kg, p.o.) significantly reversed the MK-801-induced recognition memory impairment (Figure 3A). There was no difference in total time exploring the two objects among the three groups in the training and retention sessions (training, *F*_(2,18)_ = 1.687, *p* =0.2130; retention, *F*_(2,18)_ = 1.104, *p* = 0.3529) (Figure 3B).

### 3.3. RNA Sequencing Analysis

**Figure 4:**
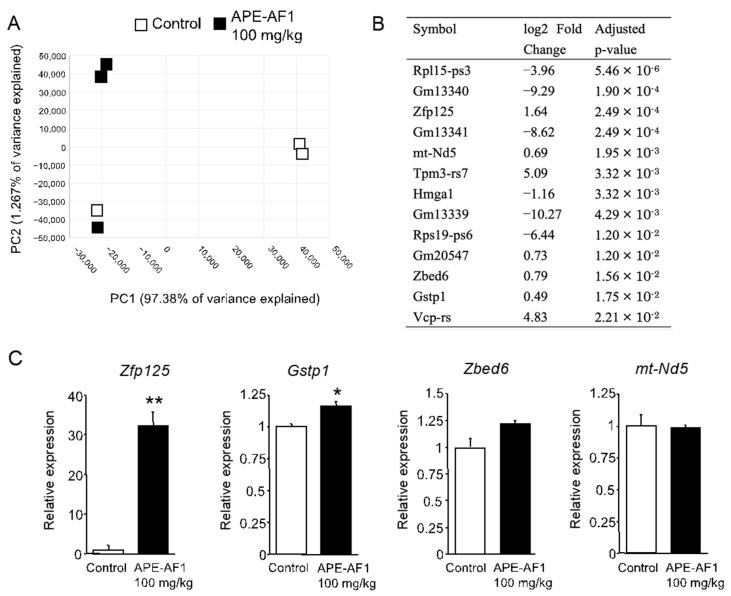
Differential expression analysis of mRNA in the hippocampus of mice treated with apple pomace extract (100 mg/kg, p.o., for 7 days). (**A**) PCA plot. (**B**) DEGs. (**C**) mRNA expression determined by real-time PCR. The real-time PCR data are shown as means ± SEM (n = 3). * *p* < 0.05, ** *p* < 0.01 vs. control.

To clarify the mechanisms by which repeated treatment with APE-AF1 (100 mg/kg, p.o.) reverses MK-801-induced memory impairment, comprehensive gene expression in the hippocampi of mice treated with APE-AF1 (100 mg/kg, p.o., for 7 days) was investigated via an RNA sequencing analysis. PCA analysis showed the differences in gene expression profiles between the two groups (Figure 4A). The differential expression analysis demonstrated a total of 13 genes to be differentially expressed in the APE-AF1-treated group compared with the vehicle-treated control (Figure 4B). We measured the mRNA expression of several genes via real-time PCR. As shown in Figure 4C, the mRNA expression of *Zfp125* and *Gstp1* was upregulated in the APE-AF1-treated group compared with the vehicle-treated control (*Zfp125*, *p* = 0.0016; *Gstp1*, *p* = 0.0385).

**Figure 5:**
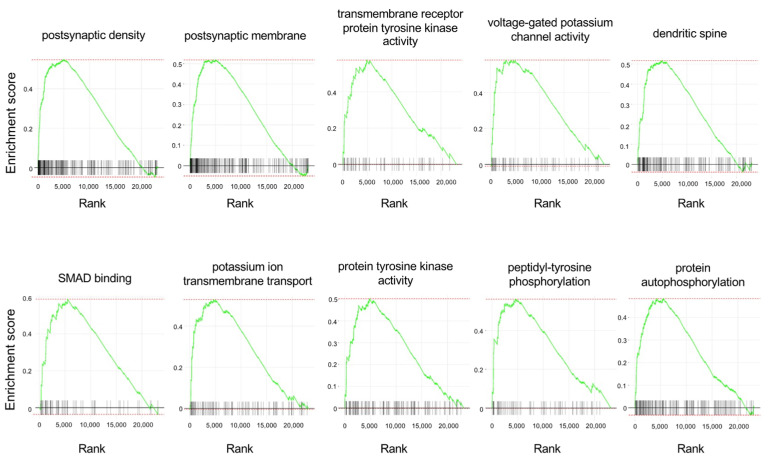
Enrichment plots of the top 10 enriched gene sets obtained using the GO database for the GSEA. Each plot shows the enrichment score against the ranked list of genes. The vertical bars on the X-axis indicate the genes belonging to the gene set.

To look for predefined gene sets that were affected by APE-AF1 treatment, we next performed a GSEA. The top 10 enriched gene sets and enrichment plots revealed by GSEA using the Gene Ontology (GO) database are shown in Table 1 and Figure 5, respectively. The top 100 GSEA results obtained using the GO database are shown in Supplementary Table S2. Notably, the GSEA revealed upregulation of multiple gene sets related to synapses, namely the “postsynaptic density”, “postsynaptic membrane”, and “dendritic spine”, in the APE-AF1-treated group compared with the vehicle-treated control.

**Figure 6:**
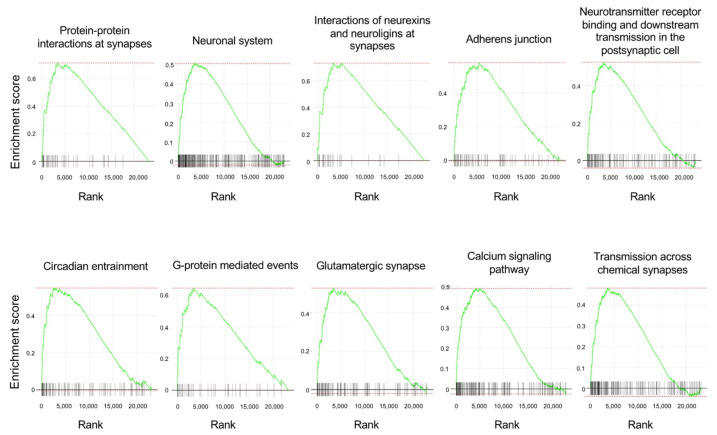
Enrichment plots of the top 10 enriched gene sets obtained using the pathway database for the GSEA. Each plot shows the enrichment score against the ranked list of genes. The vertical bars in the X-axis indicate the genes belonging to the gene set.

The top 10 enriched gene sets and enrichment plots revealed by GSEA using the pathway database are shown in Table 2 and Figure 6. The top 100 GSEA results obtained using the pathway database are shown in Supplementary Table S3. The GSEA results revealed upregulation of multiple gene sets related to synapses and neurotransmission, such as “protein–protein interactions at synapses”, “interactions of neurexins and neuroligins at synapses”, “neurotransmitter receptor binding and downstream transmission in the postsynaptic cell”, “glutamatergic synapse”, and “transmission across chemical synapses”, in the APE-AF1-treated group compared with the vehicle-treated control, consistent with the GSEA results obtained via the GO database. These results suggest that APE-AF1 altered the expression of genes related to synapses and neurotransmission.

### 3.4. The Composition of Glucosylceramide Contained in APE-AF1

The composition of glucosylceramide contained in APE-AF1 was analyzed via HPLC-ESI-Q-TOF-MS. Based on the total ion chromatogram (TIC) results, specific glucosylceramide mass spectra in an apple were scanned. As a result, glucosylceramide t18:1/16h:0 ([M + Na]^+^ = 754.545), t18:1/C20h:0 ([M + Na]^+^ = 810.607), t18:1/C22h:0 ([M + Na]^+^ = 838.638), and d18:2/C16h:0 ([M + Na]^+^ = 736.534) were detected (Supplementary Figures S2–S5). In particular, t18:1/16h:0 and d18:2/C16h:0 were detected with a strong intensity. Therefore, they were estimated to be the main glucosylceramides of APE-AF1.

## 4. Discussion

This study demonstrated that repeated treatment with APE-AF1 reverses the NMDA receptor antagonist MK-801-induced impairment of associative memory and recognition memory in mice. Since repeated treatment with APE-AF1 for 7 consecutive days before behavioral tests, but not single treatment, reverses memory impairment, we examined the comprehensive gene expression of the hippocampus in mice after repeated treatment to clarify the mechanisms of action of APE-AF1.

An RNA sequencing analysis demonstrated that repeated treatment with APE-AF1 for 7 days altered the gene expression profile in the hippocampus of mice. We showed that the mRNA expression of *Zfp125* and *Gstp1* was upregulated in the APE-AF1-treated group compared with the vehicle-treated control via real-time PCR. The *Zfp125* gene encodes a C2H2-type zinc-finger protein that is characterized as a *Foxo1*-inducible transcriptional repressor [19]. Regarding neurological diseases, *Zfp125* is a downregulated gene in the brains of prenatal excessive methionine-treated schizophrenia model mice that exhibit behavioral phenotypes reminiscent of human schizophrenia, including memory impairment [20]. GSTP1 is an isoenzyme of the glutathione-S-transferase family that catalyzes intracellular detoxifying reactions with reduced glutathione (GSH) and plays an important role in the antioxidant system [21]. It was reported that the GSTP1 levels decrease in the brains of AD patients at a severe stage [22].

Synaptic transmission using glutamate receptors is important for learning and memory. Particularly, the involvement of neurotransmission using the NMDA receptor in learning and memory has been demonstrated [23]. Activation of intracellular signaling pathways, such as extracellular-signal regulated kinase 1/2 signaling through NMDA receptor, induces gene expression associated with structural changes of the synapse, contributing to the formation of long-term memory [24]. Administration of an NMDA receptor antagonist, such as MK-801, inhibits the learning-associated activation of intracellular signaling and induces learning and memory impairments [23,25,26]. Herein, the GSEA results showed that gene sets related to synapses and neurotransmission, such as “neurotransmitter receptor binding and downstream transmission in the postsynaptic cell”, “glutamatergic synapse”, and “transmission across chemical synapses”, were upregulated after repeated treatment with APE-AF1, suggesting that APE-AF1 is likely to have improved memory by upregulating the expression of genes related to neurotransmission and synapses.

In this study, we examined the comprehensive gene expression of the hippocampus after treatment with APE-AF1 in naïve mice. The effects of APE-AF1 on the inhibition of learning-associated activation of intracellular signaling and subsequent gene expression by MK-801 should be examined in future studies. Furthermore, it is interesting to examine the memory-enhancing effects of APE-AF1 in naïve mice, because APE-AF1 upregulated the expression of gene sets related to synapse and neurotransmission in the hippocampus of naïve mice.

Our metabolomics analysis showed that the main constituents of APE-AF1 are triterpenoids and glucosylceramides (T. Narumi, J. Ichita, and K. Itoku, unpublished observation). Among triterpenoids, ursolic acid and oleanolic acid are the main constituents, consistent with previous reports [8,27]. Notably, it has been shown that ursolic acid and oleanolic acid cross the blood–brain barrier (BBB) and exhibit neuroprotective and memory-improving effects [28,29,30]. For example, treatment with ursolic acid for 11 or 15 days attenuates Aβ-induced memory impairment in mice [31,32]. A single administration of oleanolic acid reverses the memory impairment induced by scopolamine, a muscarinic acetylcholine receptor antagonist, in mice [33]. Furthermore, Jeon et al. examined the effect of oleanolic acid on synaptic transmission in hippocampal slices and showed that it facilitates hippocampal long-term potentiation, a synaptic model of memory [33].

Regarding glucosylceramide in APE-AF1, HPLC-Q-Tof-MS analysis showed 2-hydroxypalmitic acid (C16h:0) as the fatty acid, 4-hydroxy-cis-8-sphingenine (t18:1) as the sphingoid base, and glucose as the hexose (t18:1/C16h:0), consistent with previous reports [34,35]. In addition, in this study, other structurally different glucosylceramides (t18:1/C20h:0, t18:1/C22h:0, and d18:2/C16h:0) were also detected. Plant-derived glucosylceramides, which are characterized by the presence of Δ8-unsaturated long-chain bases, have attracted attention as functional food materials that may prevent AD [36]. A recent report has shown that oral administration of amorphophallus konjac extracts containing glucosylceramide, which is primarily composed of ceramide with 4,8-sphingadienine and 2-hydroxystearic acid (d18:2/18h:0), for 14 days reduces Aβ pathology and synaptic toxicity in the brains of APP transgenic mice, as well as improving memory impairment [37]. Three-month treatment with glucosylceramide from soybean, which is primarily composed of ceramide with 4,8-sphinganine and 2-hydroxypalmitic acid (d18:2/16h:0), ameliorated memory impairment in aged mice [38]. Furthermore, Eguchi et al. showed that plant ceramides can cross the BBB [39]. Collectively, the triterpenoid and glucosylceramide constituents may contribute to the memory-improving effects of APE-AF1.

## 5. Conclusions

The present study is the first to demonstrate that apple pomace extract is likely to have improved memory. We demonstrated that repeated treatment with APE-AF1 for 7 days reverses the NMDA receptor antagonist MK-801-induced impairment of associative memory and recognition memory in mice. RNA sequencing revealed that repeated treatment with APE-AF1 altered the gene expression profile in the hippocampus of mice. Furthermore, gene sets related to synapse and neurotransmission were upregulated by APE-AF1. These results suggest that the apple pomace extract may be useful for the prevention and treatment of AD.

## Figures and Tables

**Table 1:** Normalized enrichment scores (NESs) of the top 10 enriched genes sets using the GO database.

GO_ID	GO_Term	NES	Adjusted *p*-Value
GO:0014069	postsynaptic density	2.67	5.83 × 10^−3^
GO:0045211	postsynaptic membrane	2.50	5.83 × 10^−3^
GO:0004714	transmembrane receptor protein tyrosine kinase activity	2.45	5.83 × 10^−3^
GO:0005249	voltage-gated potassium channel activity	2.41	5.83 × 10^−3^
GO:0043197	dendritic spine	2.40	5.83 × 10^−3^
GO:0046332	SMAD binding	2.37	5.83 × 10^−3^
GO:0071805	potassium ion transmembrane transport	2.36	5.83 × 10^−3^
GO:0004713	protein tyrosine kinase activity	2.34	5.83 × 10^−3^
GO:0018108	peptidyl-tyrosine phosphorylation	2.32	5.83 × 10^−3^
GO:0046777	protein autophosphorylation	2.28	5.83 × 10^−3^

**Table 2:** Normalized enrichment scores (NESs) of the top 10 enriched genes sets using the pathway database.

Pathway_ID	Pathway	NES	Adjusted *p*-Value
R-MMU-6794362	Protein–protein interactions at synapses	2.63	2.84 × 10^−3^
R-MMU-112316	Neuronal system	2.48	2.84 × 10^−3^
R-MMU-6794361	Interactions of neurexins and neuroligins at synapses	2.43	2.84 × 10^−3^
mmu04520	Adherens junction	2.32	2.84 × 10^−3^
R-MMU-112314	Neurotransmitter receptor binding and downstream transmission in the postsynaptic cell	2.32	2.84 × 10^−3^
mmu04713	Circadian entrainment	2.31	2.84 × 10^−3^
R-MMU-112040	G-protein mediated events	2.29	2.84 × 10^−3^
mmu04724	Glutamatergic synapse	2.28	2.84 × 10^−3^
mmu04020	Calcium signaling pathway	2.26	2.84 × 10^−3^
R-MMU-112315	Transmission across chemical synapses	2.24	2.84 × 10^−3^

## Data Availability

Data are contained within the article and Supplementary Materials.

## Supplementary Materials

The following supporting information can be downloaded at: https://www.mdpi.com/article/10.3390/nu16020194/s1, Figure S1: Effects of a single treatment with apple pomace extract on associative memory in the passive avoidance task; Figures S2–S5: HPLC-ESI-Q-TOF-MS analysis of glucosylceramide contained in APE-AF1; Table S1: Primer sequences used for real-time PCR analysis; Table S2: Normalized enrichment scores (NES) of the top 100 enriched genes sets using the GO database; Table S3: Normalized enrichment scores (NES) of the top 100 enriched genes sets using the pathway database.
