# Supplementary material for: Apple Pomace Extract Improves MK-801-Induced Memory Impairment in Mice
Source: Nutrients. 2024 Jan 6;16(2):194. doi: 10.3390/nu16020194 (PMC10818464; doi:10.3390/nu16020194)
Supplement: Supplementary file 1 [file nutrients-16-00194-s001.zip › Supplementary Figure S2-S5.pptx]

## Slide 1
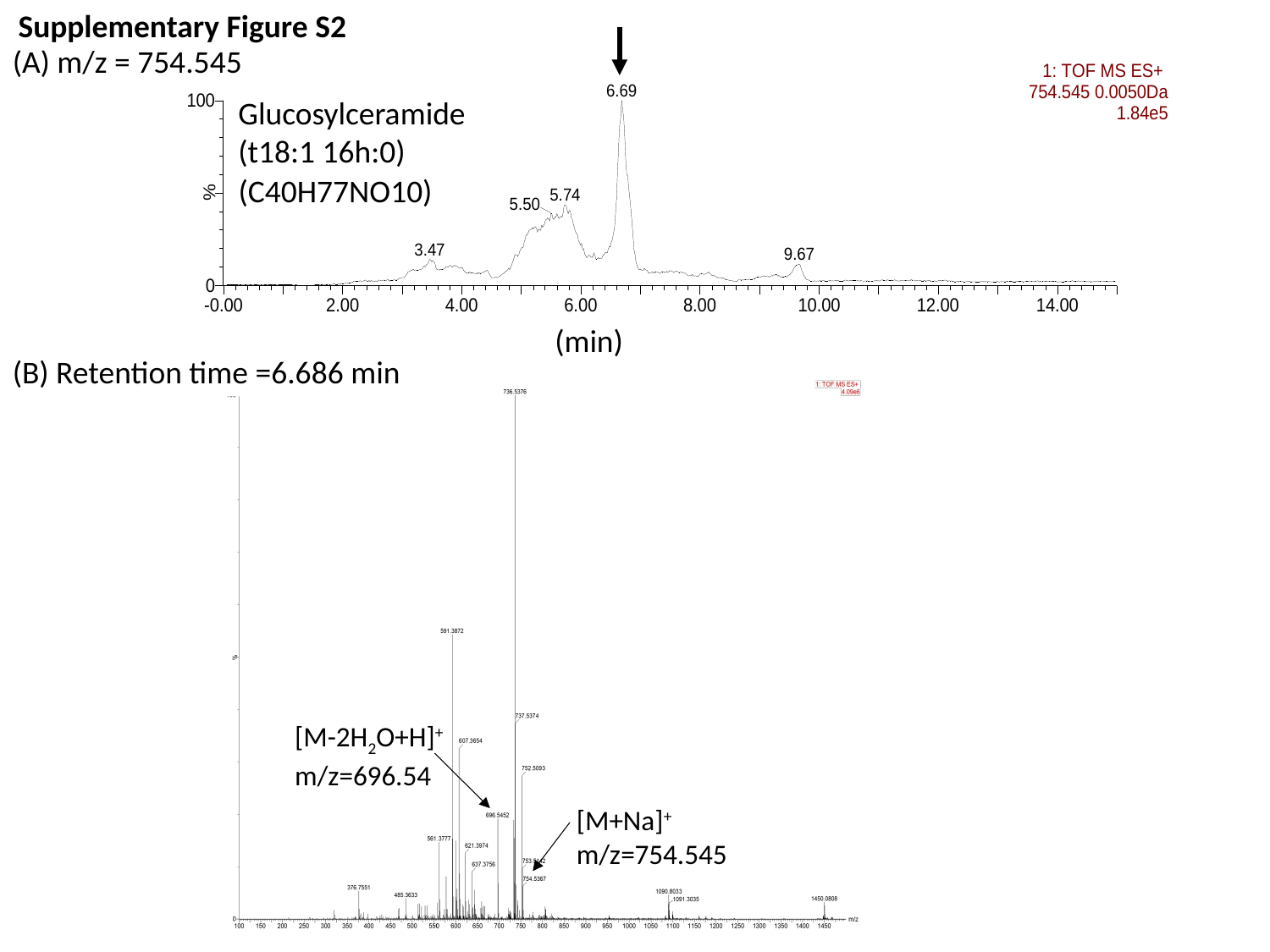

Supplementary Figure S2
(A) m/z = 754.545
Glucosylceramide (t18:1 16h:0)
(C40H77NO10)
(min)
(B) Retention time =6.686 min
[M-2H2O+H]+
m/z=696.54
[M+Na]+
m/z=754.545

## Slide 2
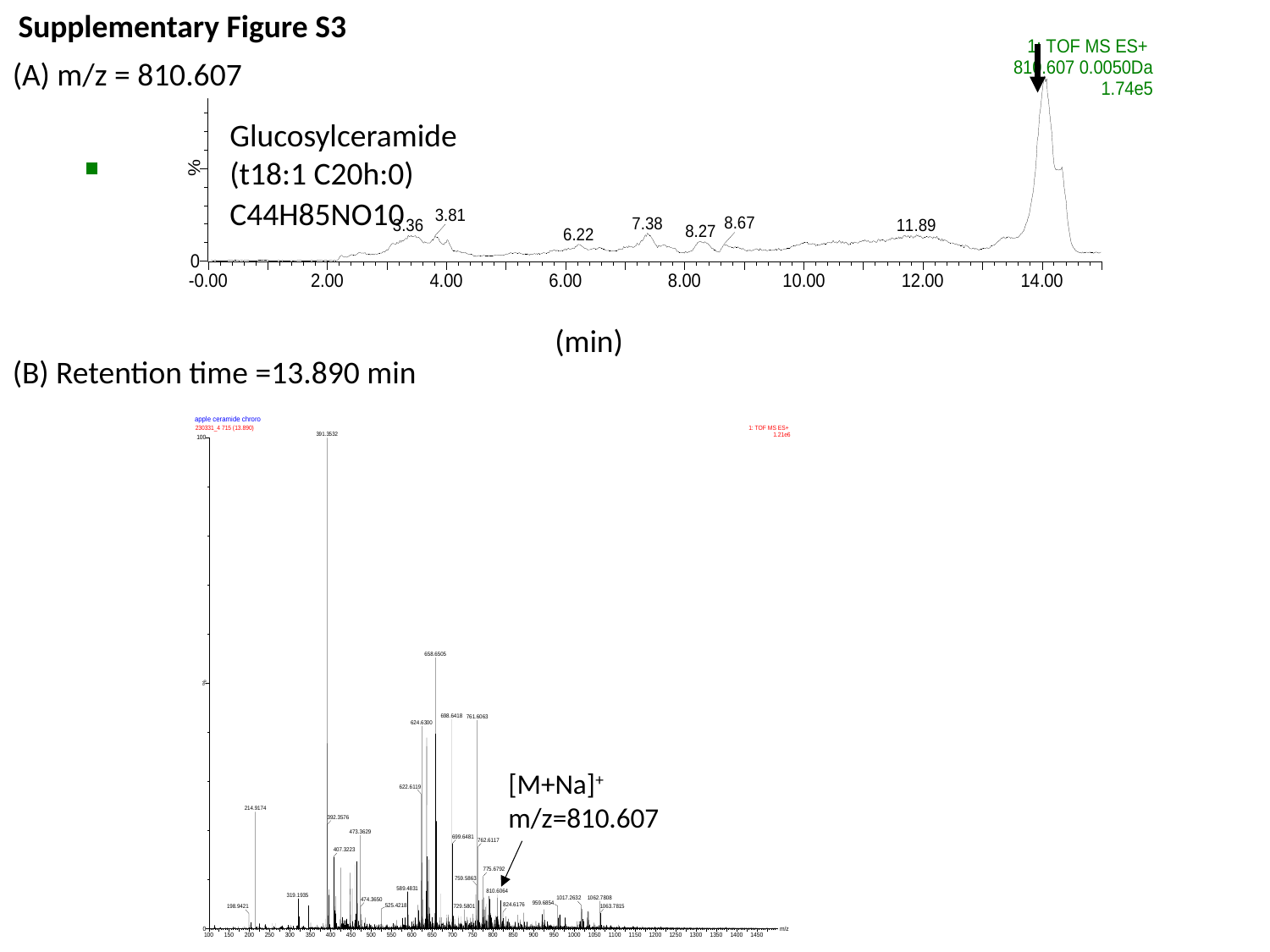

Supplementary Figure S3
(A) m/z = 810.607
Glucosylceramide
(t18:1 C20h:0)
C44H85NO10
(min)
(B) Retention time =13.890 min
[M+Na]+
m/z=810.607

## Slide 3
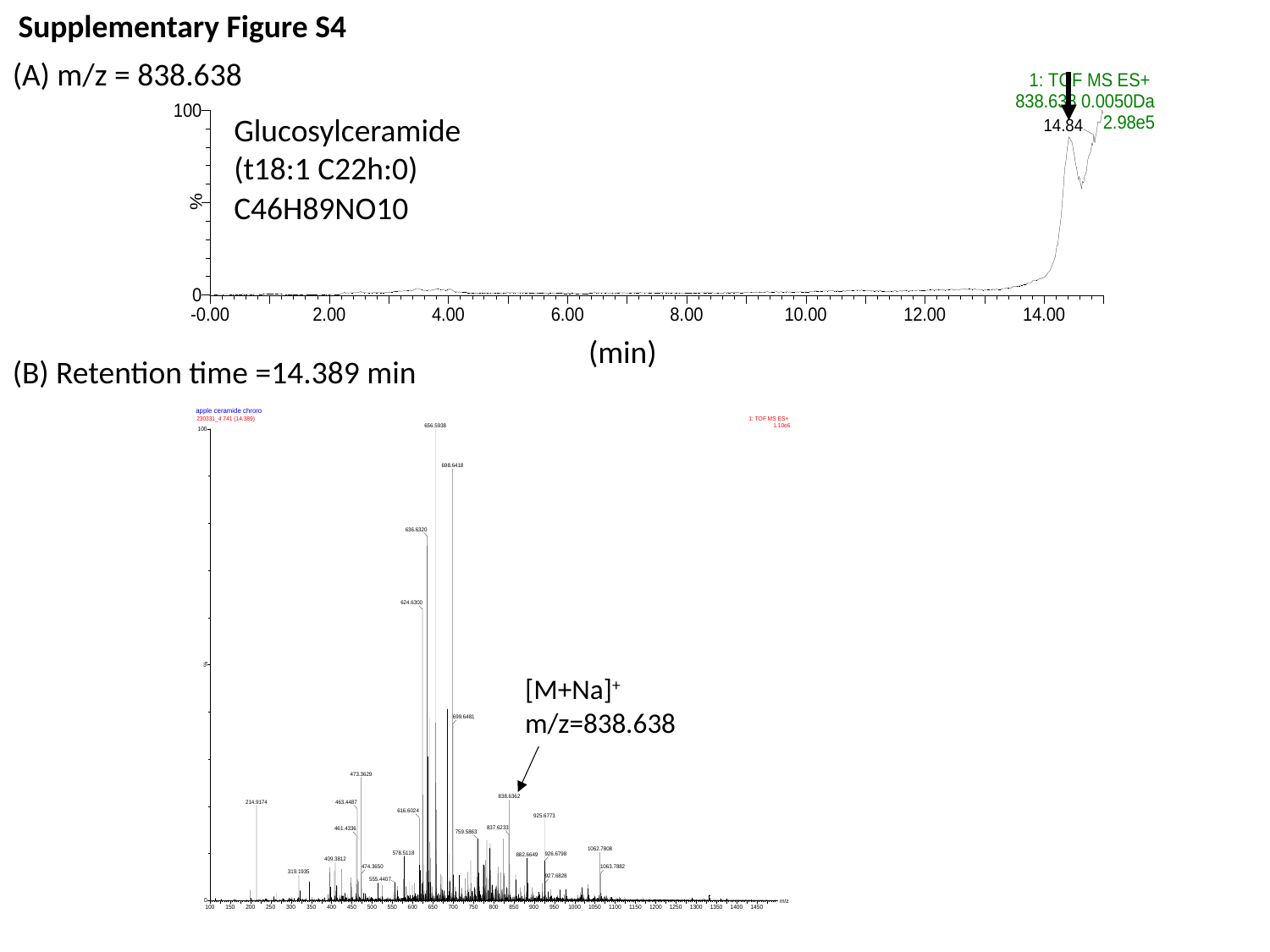

Supplementary Figure S4
(A) m/z = 838.638
Glucosylceramide
(t18:1 C22h:0)
C46H89NO10
(min)
(B) Retention time =14.389 min
[M+Na]+
m/z=838.638

## Slide 4
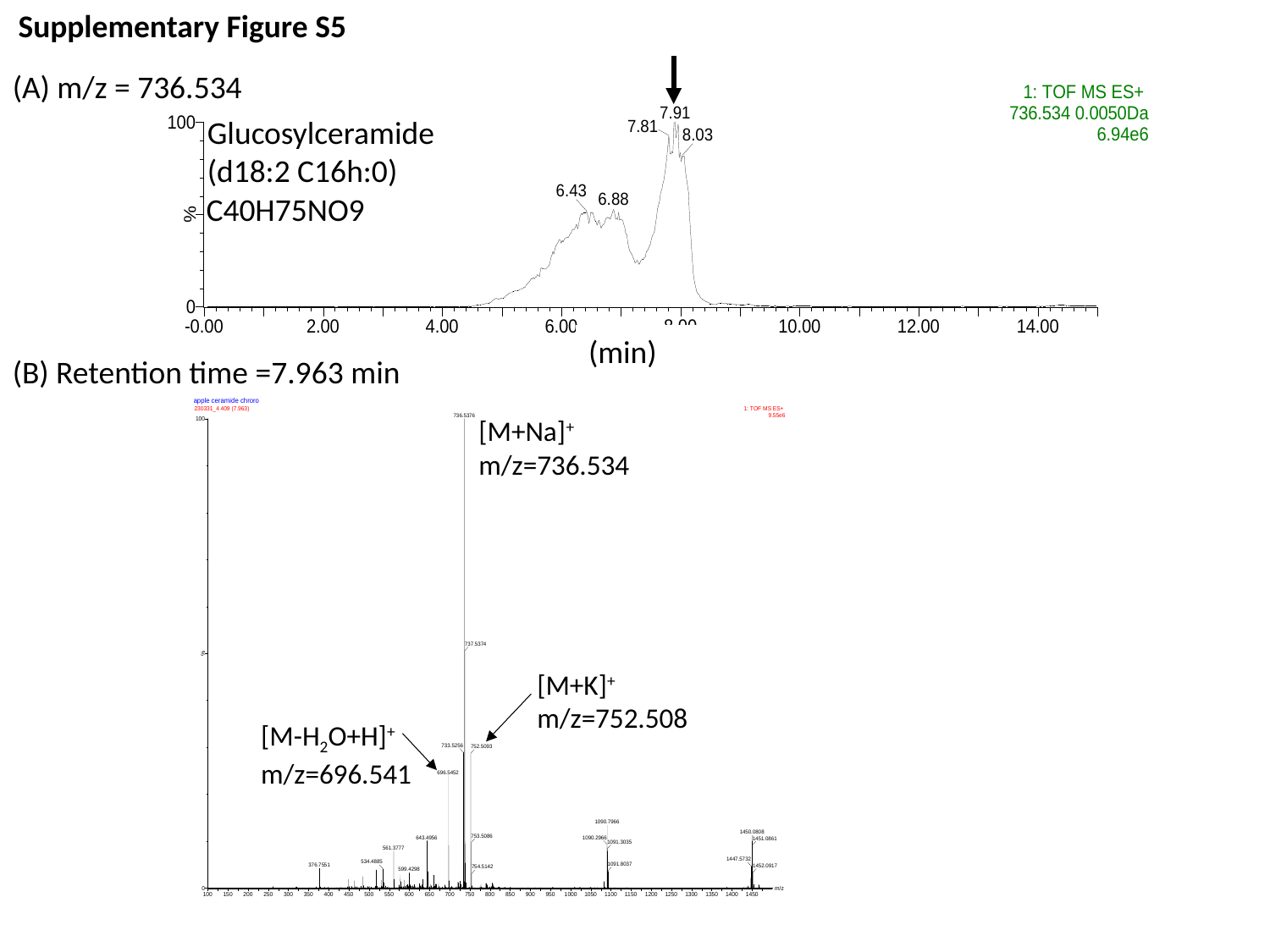

Supplementary Figure S5
(A) m/z = 736.534
Glucosylceramide
(d18:2 C16h:0)
C40H75NO9
(min)
(B) Retention time =7.963 min
[M+Na]+
m/z=736.534
[M+K]+
m/z=752.508
[M-H2O+H]+
m/z=696.541
