# Supplementary material for: Apple Pomace Extract Improves MK-801-Induced Memory Impairment in Mice
Source: Nutrients. 2024 Jan 6;16(2):194. doi: 10.3390/nu16020194 (PMC10818464; doi:10.3390/nu16020194)
Supplement: Supplementary file 1 [file nutrients-16-00194-s001.zip › Table S1.pdf]

Table S1. Primer sequences used for real-time PCR analysis

Gene	Primer Sequence	
	Forward	Reverse
Zfp125	5’-TCGTACGGGAGACATGCTATGAA-3’	5’-GAGTTCCTTAAAGAGGAAATGACCA-3’
Gstp1	5’-ATGTCACCCTCATCTACACCAAC-3’	5’-CAGGGTCTCAAAAGGCTTCA-3’
Zbed6	5’-TGCCTAGTACCAGAGCCAAAA-3’	5’-GGTTACAAATTGCTCTCCAGGT-3’
mt-Nd5	5’-GAACTTAAAATAAGCTTCAAAACTGAC-3’	5’-ATAATTGATCATGTGACAAAAAGG-3’
β-actin	5’-ACTGTCGAGTCGCGTCCA-3’	5’-GCAGCGATATCGTCATCCAT-3’
